# Real-world clinical experience with Obsidio Conformable Embolic

**DOI:** 10.1186/s42155-025-00555-w

**Published:** 2025-05-01

**Authors:** Osman Ahmed, John Karageorgiou, Abhishek Kumar, Mikin Patel, Joshua Jones, Nariman Nezami

**Affiliations:** 1 Department of Radiology, University of Chicago Medical Center, Chicago, IL USA; 2 Mallinckrodt Institute of Radiology, Washington University School of Medicine, Saint Louis, MO USA; 3 Department of Radiology, Rutgers New Jersey Medical School, Newark, NJ USA; 4 Boston Scientific Corporation, Marlborough, MA USA; 5 Division of Vascular and Interventional Radiology, Department of Radiology, MedStar Georgetown University Hospital, 3800 Reservoir Road, NW, CCC Bldg., Room CG225, Washington, DC, 20007 USA; 6 Georgetown University School of Medicine, Washington, DC, USA; 7 Lombardi Comprehensive Cancer Center, Washington, DC, USA

**Keywords:** Hemorrhage, Tumor, Bleeding, Embolization, Conformable embolic

## Abstract

**Background:**

Obsidio Conformable Embolic (Obsidio) is a ready-made hydrogel with unique shear-thinning properties, used for occlusion of blood flow to control bleeding or hemorrhage in the peripheral vasculature and embolization of hypervascular tumors. While pre-clinical and clinical data have demonstrated successful embolizations using Obsidio, clinical experience overall is still limited, prompting a multi-institutional field assessment survey to collect additional data on the clinical utility and procedural details from a variety of Obsidio users. The field survey collected data from 131 embolization procedures performed using Obsidio between May and November 2023 at 27 institutions within the United States. Data collection included embolization site, vessel size, any adjunctive embolics used. The primary objective of the survey was to evaluate technical success, defined as complete embolization of the target vasculature immediately following the index procedure, as confirmed by angiography.

**Results:**

Of the 131 embolization procedures performed, 69% (*n* = 90) were for hemorrhage control, 15% (*n* = 19) were for hypervascular tumors, and 17% (*n* = 22) were for other indications. Embolization of the gastroduodenal artery was the most common indication (*n* = 19/131; 15%). A single syringe (1 mL) or less of Obsidio was used for most cases (93%). In 33/131 cases (25%), Obsidio was combined with other embolization devices including coils (*n* = 25; 19%), particle-based embolics (*n* = 6; 4.6%), or plugs (*n* = 2; 1.5%). Technical success was achieved in 100% of Obsidio embolization cases (131/131 procedures).

**Conclusion:**

Initial clinical experience demonstrated successful embolization of end-organ bleeds and hypervascular tumors utilizing Obsidio, thus making it an effective embolic agent alone or in conjunction with other embolic devices.

## Background

**Fig. 1:**
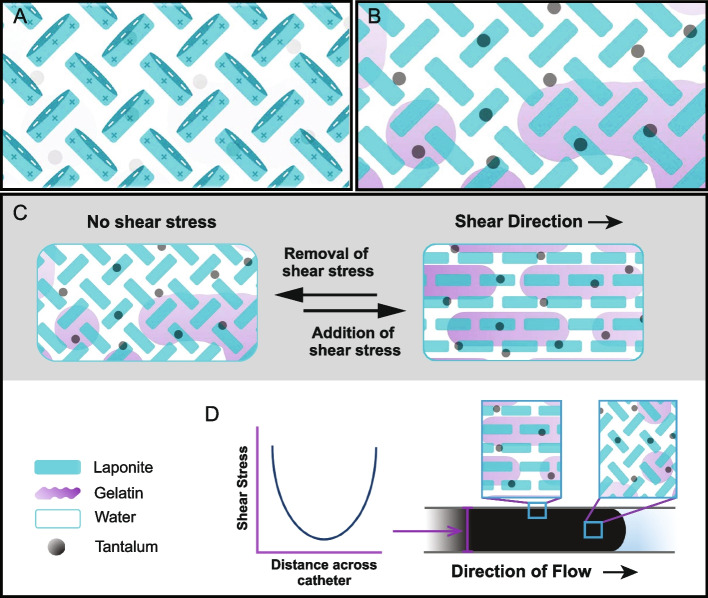
Composition of Obsidio. **A** Laponite forms a lattice structure resulting from edge-rim electrostatic interactions between neighboring particles. **B** Structural schematic with gelatin, laponite, water, and tantalum represented. **C** Schematic showing reversible changes in material structure in response to shear stress (i.e. shear thinning). **D** Diagram showing the conformational changes due to shear force. When shear force is high, the distance across the catheter is small, whereas when shear force is low, the distance across the catheter is large

Obsidio Conformable Embolic™ (Obsidio) is a new shear-thinning hydrogel which received United States (US) Food and Drug Administration (FDA) 510(k) clearance in July 2022 for the embolization of hypervascular tumors and occlusion of blood flow to control bleeding or hemorrhage in the peripheral vasculature. This novel embolic agent is comprised of four non-toxic materials: bioresorbable gelatin, laponite, tantalum, and water, which are pre-mixed and packaged as a ready-to-use hydrogel [1, 2]. Laponite is a synthetic disc-shaped nanoparticle with negative charges on the disc face and positive charges on the edge that create an electrostatic attraction between particles, forming an ordered lattice structure (Fig. 1A). Interactions between the positively charged gelatin and negatively charged laponite improve stability and help to form a hydrogel (Fig. 1B). The structure of this hydrogel creates a shear-thinning mechanism. Under high shear forces, the material structure reorients, which decreases the viscosity and allows delivery of the material through microcatheters (Fig. 1C-D). As shear forces are decreased or removed (i.e., exiting the microcatheter), the physical structure is rapidly recovered, allowing the hydrogel to conform and mechanically occlude the vessel lumen, stopping blood flow [1, 2].

Early preclinical evaluations were performed in a non-survival swine model using a prototype version of Obsidio (Ta-GEM). Embolization of the iliac artery using Ta-GEM was compared to coils in animals anticoagulated with heparin to an activated clotting time (ACT) > 300 to model patients with impaired coagulation. Embolization with Ta-GEM achieved complete and immediate focal occlusion, whereas coil embolization did not achieve hemostasis. In instances where coils failed to achieve hemostasis, subsequent Ta-GEM injection at the proximal end of the coil mass resulted in instant hemostasis and occlusion of the target artery. In a chronic swine iliac artery study, 16/16 pigs demonstrated persistent embolization with Ta-GEM up to 28 days post embolization [2]. Recent studies have demonstrated clinical success (resolution of bleeding without reintervention) in 93–100% of procedures [3–5].

In a single center prospective study enrolling four patients, Obsidio was used for the embolization of renal cell carcinoma prior to nephrectomy. The primary safety endpoint was a composite of the incidence of serious device-related adverse events, serious procedure-related adverse events, and non-target embolization. Five tumors ranging from 2.4 cm to 14.7 cm maximal diameter were embolized, and immediate occlusion of the target arteries was achieved without adverse events, satisfying the primary safety endpoint [6, 7]. Post-procedure computed tomography (CT) imaging showed no migration or recanalization, and post-resection histology (1–7 days) confirmed Obsidio within the lumen of occluded renal arteries and no evidence of recanalization.

Given the limited real-world clinical experience reported using Obsidio, a multi-institutional clinical experience survey was conducted to collect procedure details while using Obsidio, with a primary objective of evaluating technical success across a broad range of clinical applications. Herein, we summarize initial user experience, including user technique, procedural details, and clinical applications of Obsidio.

## Methods and materials

### Study design and objectives

The institutional experience survey included data from 131 embolization procedures performed using Obsidio Conformable Embolic between May and November 2023 at 21 academic and 6 community institutions within the US. Data from this field survey utilized only procedural details; no medical records were accessed; therefore, it did not require Institutional Review Board approval. Case examples followed individual institutional protocols and procedures for obtaining patient consent. The primary objective of the survey was technical success, defined as complete embolization of the target vasculature immediately following the index procedure, as confirmed by angiography.

### Obsidio Conformable Embolic

Obsidio (Boston Scientific Inc, Marlborough, MA) is available in a 1 mL syringe which is recommended to be stored between 2–8º C until the procedure. It can be removed from cold storage prior to a procedure but requires no warming or mixing before use. If the sterile barrier is not broken, the vial can be placed back in the refrigerator the same day to be used at a later date.

### Obsidio embolization techniques

The standard delivery technique for Obsidio involves navigating a microcatheter as closely as possible to the target vessel, connecting Obsidio to the catheter using a wet-to-wet technique, and delivering the material directly using steady pressure with a rate and force determined by the physician to achieve the desired level of occlusion. Once adequate vessel filling is confirmed, the microcatheter is removed. Post-embolization angiography is performed via the base catheter. In the preferred technique, prior to the Obsidio connection, the microcatheter is flushed with saline so the leading edge of Obsidio can be clearly visualized. Since Obsidio does not have issues with catheter entrapment, the catheter may be withdrawn while Obsidio is injected for embolization of longer segment vessels.

### Survey cohort and data collection

Following completion of each case, the operating physician completed a case survey reporting on technical success, vessel occlusion, procedure type, target vessel size, microcatheter information, conjunctive use of other embolic devices, and other procedural details. Physicians were asked what embolic they would have used for the procedure if Obsidio had not been available. Physicians were also asked to report any adverse events.

### Statistical analysis

Descriptive statistics (i.e., distribution/frequency) were used to summarize the available survey data.

## Results

### Obsidio embolization efficacy and indications

**Fig. 2:**
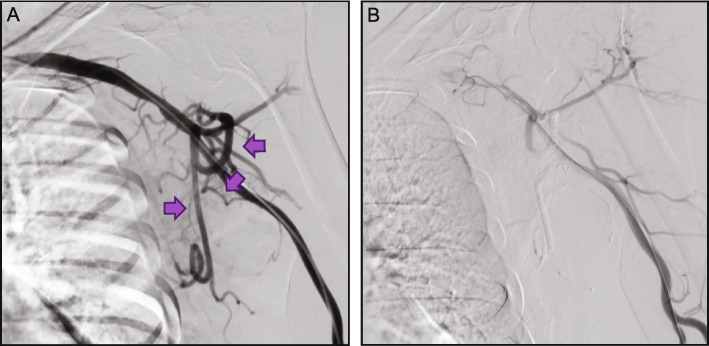
Case example 1: Preoperative tumor—scapular RCC. The patient presented with left scapular pain secondary to a metastatic RCC lesion. Prior to surgical resection, pre-operative embolization was performed. Left radial artery access was obtained, and a 4 French angled catheter was used to perform axillary angiography which showed a hypervascular mass in the left scapula with numerous feeding vessels (**A**). Obsidio embolization was performed on multiple vessels using a high flow microcatheter. Post-embolization angiogram showed markedly decreased blood flow to the hypervascular mass (**B**). The mass was successfully removed two hours after embolization with minimal intra-operative blood loss

**Fig. 3:**
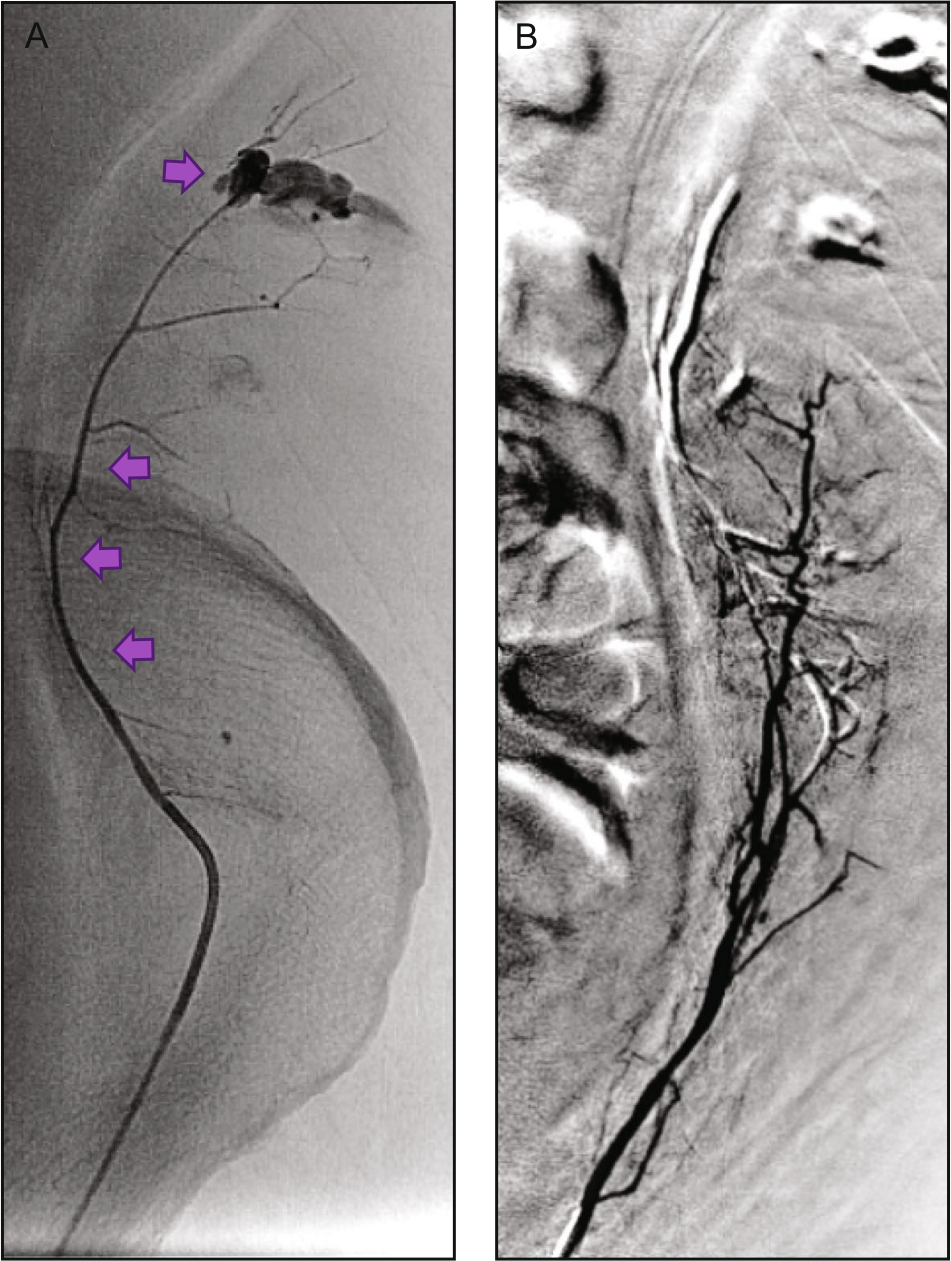
Case example 2: Trauma—circumflex iliac. The patient had ascites and presented with a post-paracentesis circumflex iliac artery bleed (**A**). Embolization with Obsidio was performed using ipsilateral common femoral access and a 4 French microcatheter. The embolized vessel had a long, single feeder, and achieved immediate stasis using 0.4 cc Obsidio for a total procedure time of less than 20 min (**B**)

**Fig. 4:**
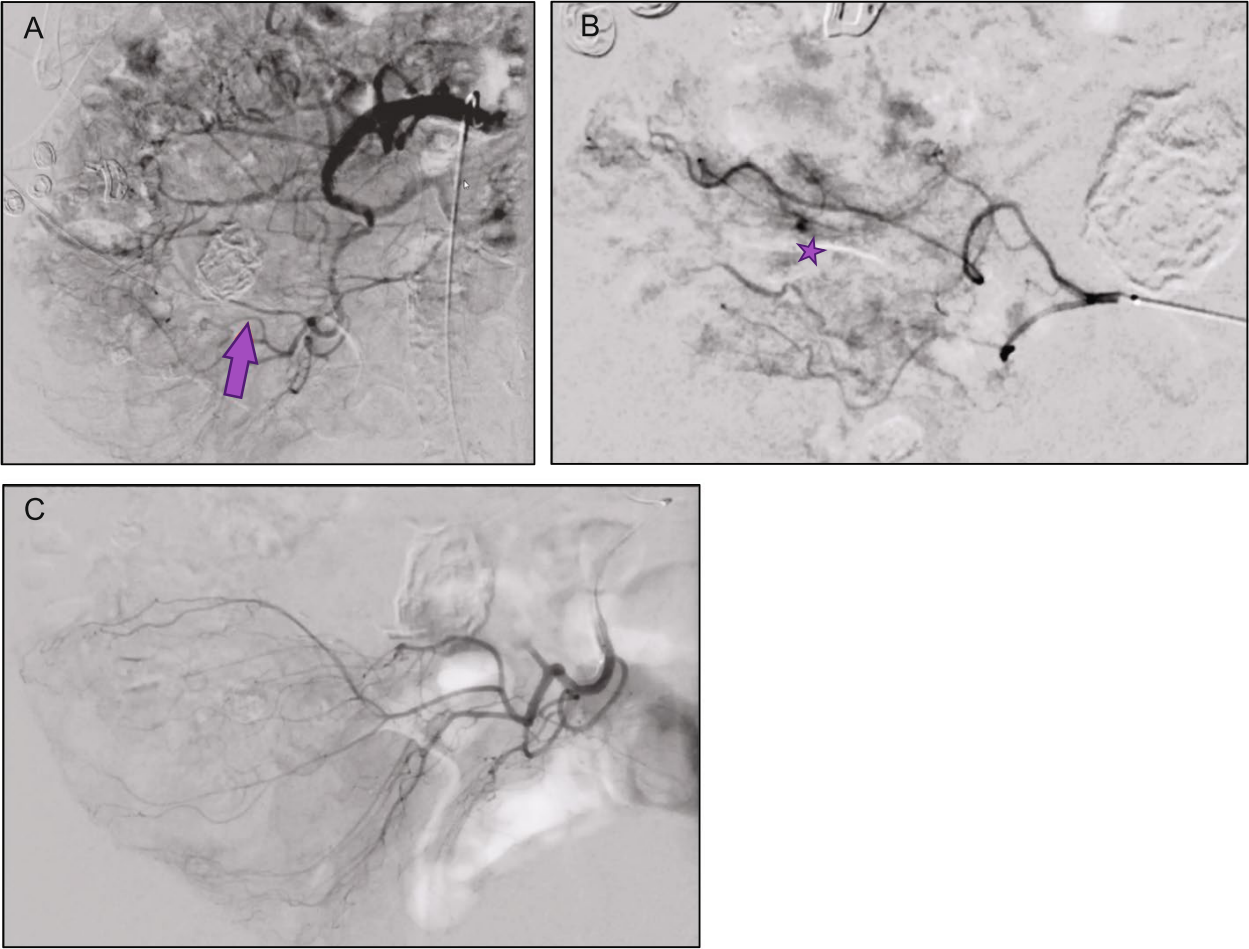
Case example 3: Lower GI bleed—pseudoaneurysm. The patient presented with hypotension and hematochezia. Emergency CT and angiograms identified a tortuous superior mesenteric artery (**A**) and small terminal branch pseudoaneurysm (PSA) (**B**). The terminal cecal branch was embolized using 0.2 cc of Obsidio, with slow injection force to allow embolic distal and proximal to the pseudoaneurysm. The PSA was completely embolized while still providing robust blood flow to the cecum (**C**)

**Fig. 5:**
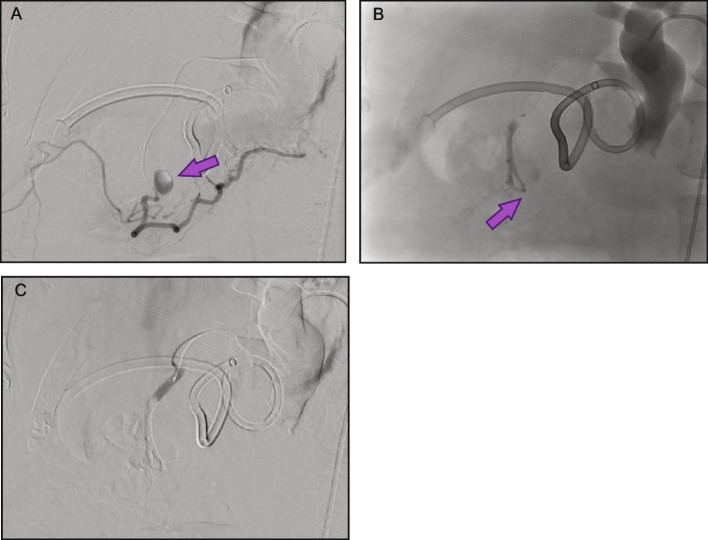
Case example 4: Upper GI bleed—GDA without coil. The patient had acute necrotizing pancreatitis and peripancreatic drain placement, and presented with hypotension, tachycardia and a GDA PSA (**A**). The GDA diameter was 3 mm and embolization was performed using 0.3 cc of Obsidio which can be seen filling the vessel (**B**). Completion angiography demonstrates complete distal embolization of the GDA and PSA (**C**)

**Fig. 6:**
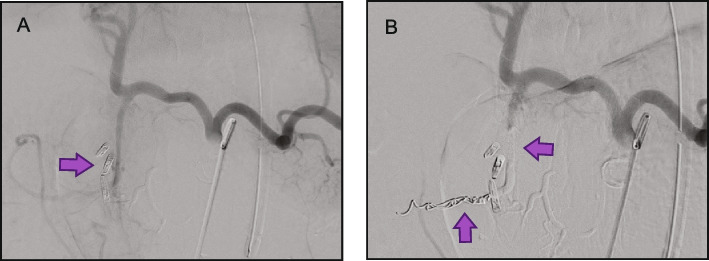
Case example 5: Upper GI bleed—GDA with adjunctive coil. The patient had a history of hepatitis C virus (HCV) cirrhosis with varices, acute drop in hemoglobin level, melena and hematemesis. Patient had extravasation from duodenal ulcer followed by clipping but did not achieve complete control, resulting in persistent GI bleeding (**A**). Given the diameter of the GDA was 5 mm and faster blood flow was anticipated, a coil was first deployed to avoid non-target embolization. Embolization was performed using 0.3 cc of Obsidio, resulting in immediate embolization and stasis (**B**)

In this survey, Obsidio achieved 100% technical success (131/131 procedures), in a variety of vessel types to stop bleeding or in hypervascular tumors, immediately following embolization as defined in the methods (Table 1). Of the 131 embolization procedures performed, 69% (*n* = 90) were for hemorrhage control, 15% (*n* = 19) in hypervascular tumors, and 17% (*n* = 22) were for other indications (Table 1). Common case examples are provided in Figs. 2, 3, 4, 5 and 6. A total of 40 different target vessels were embolized during these procedures. Within bleeding applications, the gastroduodenal artery (GDA) was the most common target vessel (*n* = 19; 21%), followed by sub-branches of renal (*n* = 14; 16%), hepatic (*n* = 10; 11%), and splenic arteries (*n* = 10; 11%). Hypervascular tumor embolizations included 9 renal angiomyolipomas (AML; 47%), 2 primary renal cell carcinomas (RCC; 11%), and 2 femoral RCC metastases (11%). A total of 4 venous applications were recorded in varices (*n* = 2) and gonadal veins (*n* = 2).

**Table 1:** Treatment location and procedure details

	**n (%)**
Embolization location	
Bleed embolization	90 (69)
Gastrointestinal	34 (38)
• Gastroduodenal artery	19 (21)
• Other	15 (17)
Renal artery	14 (16)
Hepatic artery	10 (11)
Splenic artery	10 (11)
Varices	3 (3)
Tumor embolization	19 (15)
Renal angiomyolipomas	9 (47)
Primary RCC	2 (11)
Metastatic RCC	2 (11)
Other	22 (17)
Procedure details	
Vessel diameter	
≤ 3 mm	96 (73)
> 3 mm	16 (12)
Not recorded/Unknown	19 (15)
Microcatheter internal diameter	
0.021”	33 (25)
0.027”	88 (67)
Other device (e.g. 4 F catheter, endoscope, balloon microcatheter)	8 (6.1)
Adjunctive embolization devices	33 (25)
Coils	25 (19)
Plugs	2 (1.5)
Particle-based embolics	6 (4.6)

*Abbreviations*: *F* French, *RCC* renal cell carcinoma, *n* number

### Obsidio embolization procedure technical details

The majority of target vessels were 1–3 mm in diameter (*n* = 96; 73%), in concordance with the 3 mm range specified in the Instructions for Use (IFU). Larger vessels (> 3 mm) were embolized in 16 cases (12%), with unspecified vessel size in the remaining 19 cases (15%). In 122 cases (93%), only a single syringe of Obsidio (< 1 mL) was used to complete the embolization, and a second syringe was required in only 9 cases (7%). In 33/131 cases (25%), Obsidio was combined with other embolization materials including coils (*n* = 25; 19%), particle-based embolics (*n* = 6; 4.6%), or plugs (*n* = 2; 1.5%) (Table 1). When > 3 mm vessels were targeted, coils were additionally used in 9/16 of cases (56%). High flow microcatheters (0.027” inner diameter [ID]) were the predominate delivery device (*n* = 88/131; 67%), whereas smaller 0.021” ID microcatheters (below the IFU minimum of ≤ 0.024”) were used in 33 cases (25%). Other delivery devices including 4 French catheters, endoscopes, needles, and balloon microcatheters were used in 8 cases, and the delivery device was not recorded in the remaining 2 cases (Table 1). Access was obtained through the femoral artery in 96 cases (73%), radial artery in 24 (18%), or by other routes in 11 (8%) cases.

According to the physician survey, without the availability of Obsidio, their choice of embolic device(s) (multiple answers allowed) would have been coils (*n* = 94/131; 72%), n-butyl-2-cyanoacrylate (n-BCA; *n* = 35/131; 27%), precipitating (ethylene vinyl alcohol [EVOH]-based) embolics (*n* = 19/131; 15%), and other agents (*n* = 18/131; 14%).

## Discussion

Obsidio Conformable Embolic is a new material for the embolization of bleeding or hypervascular tumors. Prior to this survey, clinical experience was limited to preoperative tumor embolization in a single center [6]. Case studies demonstrating experience with Obsidio have recently been published [3–5, 8, 9] The shear thinning mechanism of action of Obsidio is a unique characteristic among existing embolic agents, leading to handling and usage principles that differ from other types of embolics. Because of this novelty, this multi-institutional experience survey was conducted at 27 sites to better understand ease of use, preferred technique, and ideal case types, and technical success across a broad range of users.

**Fig. 7:**
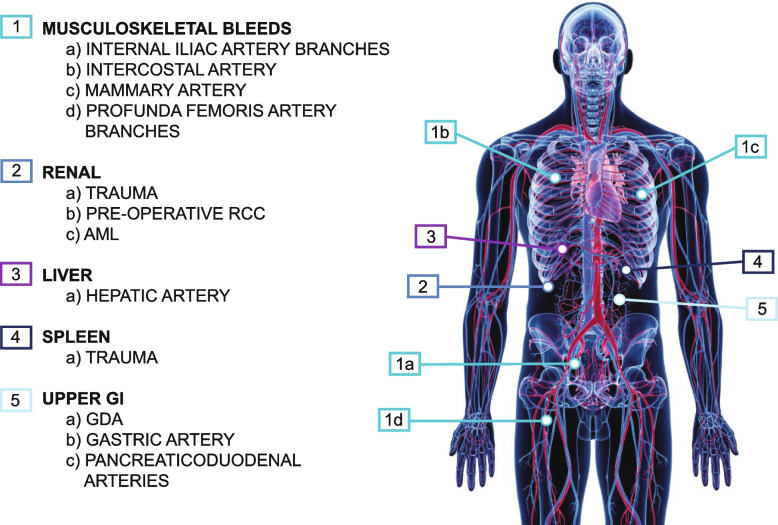
Clinical applications of Obsidio Conformable Embolic. Clinical applications include peripheral arterial bleeding and hypervascular tumors, such as musculoskeletal, renal, liver, spleen, and upper gastrointestinal bleeds

In the survey, Obsidio was an effective embolization tool, demonstrating 100% technical success, defined as complete embolization of the target vasculature immediately following the index procedure. Importantly, technical success was demonstrated alone or in combination with other embolic agents (e.g. coils) and independent of microcatheter size. Nearly 70% of the embolization procedures performed in this survey were for treatment of hemorrhage, a situation where coils are often chosen for their ability to occlude proximally with precise deployment [10, 11]. The majority of target vessels were 1–3 mm in size. Among all procedures, 93% were completed with a single syringe of Obsidio. Instances that required an additional syringe were either high volume applications (e.g. endoleak, gonadal vein) or situations where embolization of multiple supporting arteries was required. In procedures where coils were used in conjunction with Obsidio (~ 20% cases), the decision was typically dependent on the vessel size and high blood flow rate anticipated in the embolization location. In some embolizations performed near bifurcations, a coiling out technique where a coil was placed behind the Obsidio plug was used as an added safety measure. Embold coils, which are compatible with 0.027” and 0.021” microcatheters, were used in > 50% of conjunctive coil cases. A diagram showing recommended initial clinical applications for Obsidio are shown in Fig. 7. These applications include tumors and bleeds within the musculoskeletal system and end-organs, as well as upper gastrointestinal (GI) bleeds, which all have sufficient collateralization.

With additional clinical experience using Obsidio since the start of this survey, there have been complications reported when Obsidio is “aliquoted” or pushed through the catheter as a bolus followed with saline, resulting in updates to the IFU. When saline or other fluids are used to deliver Obsidio (aliquot method), mixing occurs at the interface due to turbulent flow within the hub of the catheter and radial distribution of shear stress throughout the catheter, resulting in dilution of the Obsidio material and formation of small fragments at the interface. If delivered, diluted material can behave irregularly and in an uncontrolled and unpredictable manner, potentially leading to unintended ischemia or tissue necrosis especially in anatomic structures with little vascular collateralization (e.g., lower GI). Delivery is further complicated by a rapid decrease in injection force as Obsidio is cleared from the catheter by saline. Consequently, the use of standard delivery technique is recommended when administering Obsidio. Even when using standard technique, forceful injections with saline or other fluids in or near deployed Obsidio should be avoided so as not to disrupt the blood/embolic material interface.

In clinical practice, selection of an embolic agent depends on factors such as site blood flow dynamics, size of vessel, intended duration of occlusion, desired level of occlusion, and operator preference [12–14]. Coils allow for precise deployment in a wide range of applications and vessel sizes [10, 15]. However, numerous coils can be required to achieve complete occlusion, resulting in general uncertainty with variable procedure times, costs, ability to achieve embolization, etc. [16–19]. In larger vessels (e.g. ≥ 3 mm), deployment of a single coil (or small number of coils) followed by Obsidio has been used to reduce overall number of coils or amount of time needed to achieve a high packing density with coils alone. Another major disadvantage of coils is seen in patients with underlying coagulopathy, where vessel occlusion can be difficult to achieve [20]. Occlusion with Obsidio does not depend on patients having an intact intrinsic coagulation cascade, and thus may provide an advantage in this patient population. Further evaluation treating this patient population with Obsidio is needed [1, 2].

Liquid embolics can provide rapid embolization, but require long mixing times, can result in catheter entrapment, and can be more difficult to control which can result in non-target embolization [10, 15]. Glues, primarily n-BCA, polymerize rapidly. Lipiodol is mixed with n-BCA at varying ratios to provide radiopacity and slow polymerization. Careful handling is required during mixing and delivery to avoid contact with blood or saline including use of specialized syringes and connectors as well as flushing the catheter with D5W (dextrose 5% in water). Following injection, the catheter must be quickly withdrawn to avoid catheter entrapment. EVOH-based embolics are dissolved in dimethyl sulfoxide (DMSO) and undergo precipitation upon mixing with aqueous systems, such as blood. Because of the DMSO solvent, these agents must be injected slowly (≤ 0.3 mL per minute) to avoid vasospasm and pain during delivery. Furthermore, these agents require a DMSO compatible microcatheter and a DMSO preflush to avoid clogging. In contrast, Obsidio is a premixed water-based hydrogel that does not undergo precipitation or polymerization, avoiding material compatibility problems, specialized handling requirements, and flow rate limitations. When delivered through a microcatheter, shear forces reorient the gel causing a drop in viscosity. Upon exiting the microcatheter, this process is rapidly reversed as the structure reforms without an intervening phase change, thus Obsidio tends to provide more proximal occlusion than traditional liquid embolics. Whereas embolization with liquid embolics is typically performed in < 1 mm vessels [10, 12, 13, 21], embolization with Obsidio was primarily performed in vessels 1–3 mm in diameter. Physicians accustomed to liquid embolics should be aware that Obsidio behaves counterintuitively with respect to injection rate and distal travel. With Obsidio, faster injection rates produce greater shear stress which further reduces the material’s viscosity. The low viscosity allows it to spread out quickly as it leaves the catheter, contact the vessel wall, and form a plug-like configuration resulting in proximal occlusion. Conversely, a slower injection results in less shear force, which causes the material to extrude out of the microcatheter and be carried more distally until it reaches a vessel tapering to form an occlusion. Given the unique properties of Obsidio, delivery can be paused and restarted prior to withdrawing the catheter, without risk of entrapment. A case series with 11 Obsidio procedures by Pal et. al. reported an average distal penetration of 24 mm (measured linearly from the catheter tip) representing a relatively proximal occlusion, but with a range of 1–44 mm highlighting the influence of technique and flow dynamics [4]. Overall, the unique properties of Obsidio result in rapid, controlled, permanent occlusion, independent of coagulation status and without catheter entrapment, making it an ideal material for a broad range of embolization procedures [1].

The real-world evidence of Obsidio presented herein is limited by the reliance on the submission of completed case report surveys by treating physicians, the off-label use of Obsidio in some cases, the non-mandated collection of adverse event data, and the lack of defined post-treatment follow-up. However, results are consistent with those of the first-in-human study and provide useful insights into initial real-world user experience and applications of Obsidio [6, 7]. To further evaluate safety, effectiveness (technical and clinical success), in addition to other endpoints related to patient selection, a prospective multi-center registry study (OCCLUDE) is ongoing and collecting real-world evidence data on patients treated in the US with Obsidio Conformable Embolic (NCT 06170619).

## Conclusions

Overall, initial clinical experience with Obsidio Conformable Embolic demonstrated it to be an effective and reliable therapy that can be used in a variety of embolization procedures. Embolization using Obsidio achieved 100% technical success within this survey. A prospective multicenter registry is currently underway to further evaluate safety and effectiveness of Obsidio in real-world clinical practice.

## Data Availability

All data generated or analyzed from the physician surveys are included in this article. Patient or procedure data included in the case studies beyond what is provided will not be shared.

## Abbreviations

ACT: Activated clotting time
AML: Angiomyolipoma
CT: Computed tomography
D5 W: Dextrose 5% in water
DMSO: Dimethyl sulfoxide
EVOH: Ethylene vinyl alcohol
FDA: Food and Drug Administration
GDA: Gastroduodenal artery
GI: Gastrointestinal
ID: Inner diameter
IFU: Instructions for Use
HCV: Hepatitis C virus
LME: Limited market evaluation
n-BCA: N-butyl-2-cyanoacrylate
Obsidio: Obsidio Conformable Embolic
PSA: Pseudoaneurysm
RCC: Renal cell carcinoma
US: United States of America
